# Neural substrates underlying motor skill learning in chronic hemiparetic stroke patients

**DOI:** 10.3389/fnhum.2015.00320

**Published:** 2015-06-03

**Authors:** Stéphanie Lefebvre, Laurence Dricot, Patrice Laloux, Wojciech Gradkowski, Philippe Desfontaines, Frédéric Evrard, André Peeters, Jacques Jamart, Yves Vandermeeren

**Affiliations:** ^1^Institute of Neuroscience, Université Catholique de LouvainBrussels, Belgium; ^2^Neurology Department, CHU Dinant-Godinne UCL Namur, Université Catholique de LouvainYvoir, Belgium; ^3^ImagilysBrussels, Belgium; ^4^Faculty of Electronics and Information Technology, Institute of Radioelectronics, Warsaw University of TechnologyWarsaw, Poland; ^5^Neurology Department, Site Saint-Joseph, CHCLiège, Belgium; ^6^Neurology Department, Clinique Saint-PierreOttignies, Belgium; ^7^Service de Neurologie, Unité Neuro-Vasculaire, Cliniques Universitaires Saint Luc UCL, Université Catholique de LouvainBrussels, Belgium; ^8^Scientific Support Unit, CHU Dinant-Godinne UCL Namur, Université Catholique de LouvainYvoir, Belgium; ^9^Louvain Bionics, Université Catholique de LouvainLouvain-la-Neuve, Belgium

**Keywords:** motor skill learning, stroke, fMRI, neurorehabilitation, premotor cortex, resting-state fMRI, functional connectivity

## Abstract

Motor skill learning is critical in post-stroke motor recovery, but little is known about its underlying neural substrates. Recently, using a new visuomotor skill learning paradigm involving a speed/accuracy trade-off in healthy individuals we identified three subpopulations based on their behavioral trajectories: fitters (in whom improvement in speed or accuracy coincided with deterioration in the other parameter), shifters (in whom speed and/or accuracy improved without degradation of the other parameter), and non-learners. We aimed to identify the neural substrates underlying the first stages of motor skill learning in chronic hemiparetic stroke patients and to determine whether specific neural substrates were recruited in shifters versus fitters. During functional magnetic resonance imaging (fMRI), 23 patients learned the visuomotor skill with their paretic upper limb. In the whole-group analysis, correlation between activation and motor skill learning was restricted to the dorsal prefrontal cortex of the damaged hemisphere (DLPFC_damh_: *r* = −0.82) and the dorsal premotor cortex (PMd_damh_: *r* = 0.70); the correlations was much lesser (−0.16 < *r* > 0.25) in the other regions of interest. In a subgroup analysis, significant activation was restricted to bilateral posterior parietal cortices of the fitters and did not correlate with motor skill learning. Conversely, in shifters significant activation occurred in the primary sensorimotor cortex_damh_ and supplementary motor area_damh_ and in bilateral PMd where activation changes correlated significantly with motor skill learning (*r* = 0.91). Finally, resting-state activity acquired before learning showed a higher functional connectivity in the salience network of shifters compared with fitters (qFDR < 0.05). These data suggest a neuroplastic compensatory reorganization of brain activity underlying the first stages of motor skill learning with the paretic upper limb in chronic hemiparetic stroke patients, with a key role of bilateral PMd.

## Introduction

Stroke is a devastating disorder that causes life-long upper limb hemiparesis in 30–70% of survivors (Lai et al., 2002; Kwakkel et al., 2003). The biochemical mechanisms triggered by acute stroke (e.g., edema resolution, inflammation, up- and down-regulation of neurotransmitters) play a prominent role in early recovery (Kreisel et al., 2006; Carey and Seitz, 2007). Beyond these biochemical cascades, recovery of motor function also relies on neuroplastic reconfiguration of the cortical motor network and its descending projections, which support transfer of impaired functions toward undamaged areas of the brain (Feydy et al., 2002; Johansen-Berg et al., 2002; Lotze et al., 2006; Lindenberg et al., 2010; Schulz et al., 2012). Although this neuroplastic reorganization may reflect a simple re-routing of information flow through pre-existing, undamaged pathways, stroke patients must learn how to recruit these neuronal resources. To some extent, recovering from hemiparesis might be conceptualized as a particular form of motor skill learning, in other words, learning to use the reconfigured motor network to optimize planning, execution and movement control of the paretic upper limb. Indeed, the idea that motor skill learning plays a central role in post-stroke motor recovery is becoming a major focus in neurorehabilitation (Matthews et al., 2004; Krakauer, 2006; Dipietro et al., 2012; Kitago and Krakauer, 2013).

The neural substrates of motor skill learning are relatively well elucidated in healthy individuals. Functional magnetic resonance imaging (fMRI) studies demonstrated that motor skill learning relies on a network encompassing the primary motor cortex (M1), supplementary motor area (SMA), premotor cortex (PM), dorsolateral prefrontal cortex (DLPFC), cerebellum and basal ganglia (Ghilardi et al., 2000; Halsband and Lange, 2006; Debas et al., 2010; Hardwick et al., 2013). Recently, the definition of motor skill learning has been refined to a training-induced acquisition and improvement of motor performance (i.e., skills), persisting over time and characterized by a shift of the speed/accuracy trade-off (SAT), automatisation and reduction of performance variability (Reis et al., 2009; Dayan and Cohen, 2011; Krakauer and Mazzoni, 2011). Using a visuomotor skill learning paradigm involving a SAT, we demonstrated that different that different behavioral trajectories can be observed in healthy individuals during the first stages of motor skill learning (Lefebvre et al., 2012). The first one was characterized either by improvement in both speed and accuracy or by improvement of one parameter without a concomitant worsening of the other one; this resulted in a shift of the SAT, which suggests a rapid and successful motor skill learning. The second behavioral trajectory was characterized by opposite changes in speed and accuracy over time, resulting in less efficient motor skill learning. E.g., when speed improved, accuracy worsened, resulting in slight improvement in the SAT. Finally, the third behavioral trajectory was characterized by a deterioration of both speed and accuracy or a lack of any improvement. According to their behavioral trajectory, the subjects were refereed as shifters, fitters and non-learners respectively. These different behavioral trajectories were observed despite identical instructions and experimental conditions, and they were associated with specific brain activation patterns. Specifically, in the efficient shifters, activation was found in the M1, cerebellum and the SMA where the activation changes correlated with performance improvement, suggesting that the SMA plays a key role in early motor skill learning involving a SAT. In the less efficient fitters, there was only a non-significant correlation in the cerebellum (Lefebvre et al., 2012).

In stroke patients, functional brain imaging has been used extensively to explore the reorganization of the network controlling the paretic arm or hand. Grossly, early after stroke, this reorganized network is characterized by compensatory recruitment of the undamaged hemisphere, especially the motor and premotor areas (Feydy et al., 2002; Tombari et al., 2004; Jaillard et al., 2005; Ward and Frackowiak, 2006) and/or widespread activation in the damaged hemisphere with extensive activation of the somatosensory and premotor areas (Tombari et al., 2004; Ward et al., 2006). Over time, motor recovery is associated with a shift of activation back toward the damaged hemisphere (Pineiro et al., 2001; Jaillard et al., 2005; Favre et al., 2014; Grefkes and Ward, 2014) and a progressive recruitment of the cerebellum ipsilateral to the paretic hand (Small et al., 2002). In addition, changes in brain connectivity have been associated with motor function recovery after stroke (Schaechter et al., 2009; Jiang et al., 2013; Grefkes and Ward, 2014). Early after stroke, both anatomical and functional connectivity (FC) decrease within the damaged hemisphere; over time, motor function recovery is associated with gradual recovery of connectivity (Pannek et al., 2009; Westlake et al., 2012; Golestani et al., 2013). Thus, the more similar the reorganized motor network becomes to that of healthy individuals, the better the recovery. Nevertheless, the undamaged hemisphere may still play a vicarious role in recovered motor control of the paretic hand (Johansen-Berg et al., 2002; Werhahn et al., 2003; Tombari et al., 2004; Lotze et al., 2006; Bestmann et al., 2010; Grefkes and Ward, 2014). It has also been suggested that the resting-state FC correlates with the motor recovery potential (Park et al., 2011, 2014; Yin et al., 2012; Golestani et al., 2013; Ovadia-Caro et al., 2013; Dacosta-Aguayo et al., 2014).

Since functional reorganization occurs in the network supporting motor recovery of the paretic upper limb after stroke, it seems logical that similar neuroplasticity should occur in the network underlying motor skill learning. However, despite extensive fMRI studies of the functional neuroanatomy of motor skill learning in healthy individuals (Ghilardi et al., 2000; Halsband and Lange, 2006; Debas et al., 2010; Lefebvre et al., 2012; Hardwick et al., 2013), very few studies have assessed stroke patients. Using a region of interest (ROI) approach, one study with 10 chronic stroke patients performing visuomotor tracking with the paretic hand showed a bilaterally reorganized pattern with a predominance in the undamaged hemisphere during the pre-training fMRI session (Carey et al., 2002). After training, activation was partially transferred back toward the damaged hemisphere, suggesting functional reorganization (Carey et al., 2002). Another study using ROI showed decreased task-related fMRI activation in the contralesional M1 of nine chronic stroke patients after 3 days of training on a serial targeting task (Boyd et al., 2010). A recent fMRI study highlighted the differences in brain activation patterns between nine healthy individuals and nine chronic stroke patients during training over several days on an implicit sequential visuomotor tracking task (Meehan et al., 2011). Compared with healthy individuals, motor skill learning and retention in stroke patients relied on a reorganized network involving compensatory activations, especially in prefrontal attentional areas such as the DLPFC. Finally, during baseline performance of a sequential grip-force tracking task, 10 chronic stroke patients showed reduced fMRI activation in the damaged hemisphere compared with healthy controls (Bosnell et al., 2011). After repeated training, fMRI activation decreased in healthy controls but was maintained or increased in stroke patients.

These four studies involved relatively small cohorts of mostly high-functioning patients, typically with sub-cortical strokes, and they did not characterize motor skill learning through SAT. Instead, they compared fMRI activation related to motor performance pre- and post-training, and two used an ROI approach (Carey et al., 2002; Boyd et al., 2010). Since motor learning plays a key role in motor function recovery, better knowledge of the neurophysiology of motor skill learning after stroke should lead to the refinement of recovery models and translate into the development of specific neurorehabilitation methods based on the principles of motor learning.

Motor skill learning can be divided in two stages: a fast on-line learning process leading to large performance improvement over a single training session (i.e., early stages of motor skill learning as described is the present study), and a slower process involving smaller performance gains obtained through repeated training sessions (Dayan and Cohen, 2011).

This study aimed to specifically explore the early stages of motor skill learning with the paretic hand in chronic hemiparetic stroke patients, using an innovative motor skill learning paradigm with a SAT. This is a first step to understand the “recovery process” in stroke patients, whether residual motor learning aptitudes are present and which brain areas are (neuroplastically?) involved. A better knowledge about motor (skill) learning in stroke patients could help to refine neurorehabilitation protocols, in which motor learning is often imbedded as an implicit assumption but poorly recognized. The purposes of this study were: (i) to use random effect (RFX) analyses of whole-brain fMRI activation to identify the neural substrates underlying the first stages of motor skill learning involving a SAT in a larger cohort of chronic stroke patients using their paretic upper limb, (ii) to determine whether shifter and fitter stroke patients recruit specific neural substrates, and (iii) to determine whether resting-state FC acquired before training would predict the behavioral trajectory (shifter/fitter) during the first stage of motor skill learning and/or correlate with the amount of motor skill learning.

## Material and methods

### Population

**Figure 1:**
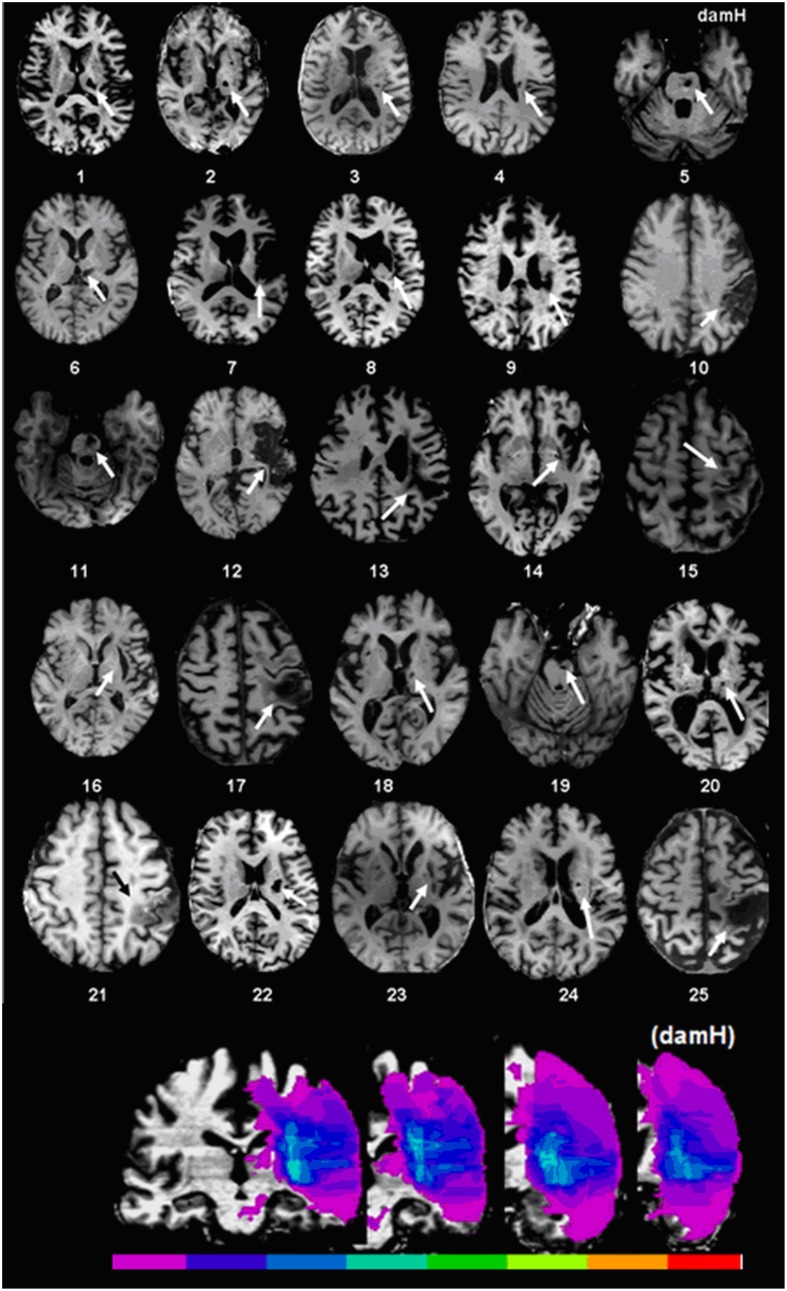
**Stroke localization and overlap. Upper panel:** T1 magnetic resonance imaging (MRI) at the level of the main stroke injury. **Lower panel**: Lesion overlap in stroke patients symbolized by the color scale. Purple represents the stroke area of a single patient, green represents localization shared by half of the patients, and red indicates localization shared by all of the patients. For patients with lesions on the right side of the brain, the 3D-T1 MRI was flipped. The map of lesion localization and overlap was created with MRIcro 1.4. DamH: damaged hemisphere.

The experimental protocol was approved by the local Ethical Committee (CHU Dinant-Godinne, UCL) and was conducted according to the recommendations of the Helsinki declaration. After providing written informed consent, 25 chronic stroke patients meeting the following criteria were selected: *Inclusion*: (i) being a chronic (>6 months) stroke patient aged 18-90 years, (ii) presenting a chronic motor deficit in the upper limb (i.e., chronic hemiparesis), (iii) having a vascular brain lesion demonstrated by cerebral imaging (Figure 1); *Exclusion*: (i) being unable to perform the task or to understand instructions, (ii) presence of intracranial metal, (iii) alcoholism, (iv) pregnancy, (v) cognitive impairment or psychiatric disorder, (vi) any contraindication to MRI. The following measures were assessed: disability with the modified Rankin Scale (mRS), level of impairment with the National Institutes of Health Stroke Scale (NHISS), residual dexterity with the Purdue Pegboard Test (PPT), maximal hand force (MaxHF) with a whole-hand Jamar dynamometer and manual ability with the ABILHAND scale (Penta et al., 2001) (Table 1). Patients #12, 13, and 15 had participated in a previous study at least 1 year before, exploring motor skill learning enhancement by transcranial direct current stimulation (tDCS) (Lefebvre et al., 2013). They were included in the present study because this circuit was different from those used in the previous experiment, so they learned from scratch a new circuit, as did the other patients. These three patients showed no significant difference from the other patients on (i) their new baseline motor performances and (ii) their new performance evolution (Table 2). This analysis was performed using the Crawford and Howell statistical test to compare an individual score to a small population (Crawford and Howell, 1998; Crawford and Garthwaite, 2002). Additional fMRI analyses did not demonstrate difference with the other patients (Supplementary Figure 1 and Supplementary Table 1).

**Table 1:** **Clinical characteristics**.

**Patients**	**Gender**	**Age**	**Time since stroke (years)**	**Main stroke lesion**	**Dominant hand**	**Paretic hand**	**Paretic hand PPT (n)**	**Non-paretic hand PPT (n)**	**Paretic hand MaxHF (Kg)**	**Non-paretic hand MaxHF (Kg)**	**ABILHAND (logits)**	**mRS**	**NIHSS**
1	F	66	14	SC	R	R	11.7	14.3	30	30	4.4	1	1
2	M	60	9	SC	R	R	4.3	9	27	33	0.4	3	4
3	M	68	10	SC	R	R	9	7.3	44	33	2.5	2	2
4	M	71	4	SC	R	R	10.6	12.7	22	31	1.7	3	3
5	M	61	2	SC	R	R	11	13	43	57	6	1	1
6	M	64	8	SC	L	L	2	14.7	42	46	0.3	3	4
7	M	58	0.6	C	R	R	3.3	13	19	46	0.4	3	7
8	M	53	3	C	L	R	11.3	12.7	45	35	3.2	1	1
9	F	72	2	SC	R	L	7.3	11	8	21	0.3	2	2
10	M	53	0.5	C	R	R	6.3	14.7	29	44	−0.1	3	4
11	M	63	10	SC	R	L	1.7	8.3	23	47	1.3	1	1
12	M	65	3	C	R	L	3	13.7	26	35	1.9	2	4
13	M	50	5	C	R	L	7	16.3	28	47	1.7	2	2
14	F	68	15	C	R	R	9	12.3	32	34	1.8	2	2
15	M	57^*^	3	C	R	R	9	11.7	55	50	1.9	2	2
16	M	69	3	C	R	R	10	9.7	44	39	1.7	1	0
17	M	57^*^	8	C	R	L	0.3	11	12	37	−0.5	2	/
18	M	82	3	SC	R	L	9	13.7	37	38	3.8	2	2
19	M	74	2	SC	R	R	5.6	9	30	36	2.4	2	2
20	M	62	10	SC	R	R	11	12	44	42	3.8	2	2
21	F	66	2	C	R	R	0	/	0	/	/	3	8
22	M	45	0.6	SC	R	R	8	12	21	41	−0.4	2	4
23	F	72	4	SC	R	L	2.3	8	11	23	−0.3	4	3
24	M	75	0.5	SC	R	R	0	11.3	20	43	−1.0	3	4
25	M	75	4	C	R	L	8.7	12.7	41	44	2.7	2	0
Mean ± SD	5F/20M	64 ± 9	4.9 ± 4.3	14SC/11C	23 R/2L	16 R/9L	6.7 ± 3.8	11.8 ± 2.4	31 ± 12	39 ± 9	1.8 ± 1.7	2.2 ± 0.8	2.7 ± 2.0

*M, male; F, female; SC, subcortical stroke; C, cortical stroke; R, right; L, left; PPT, Baseline Purdue Pegboard Test score; n, number of pegs inserted in 30 s (mean of three trials); MaxHF, Maximal hand grip force; Kg, kilograms; mRS, modified Rankin Scale; NIHSS, National Institute of Health Stroke Score; /, missing data*.

* *Patients with a partial lesion of M1*.

**Table 2:** **Comparison of behavioral performances**.

**Stroke patients**	**Baseline PI**	***t*-value**	***p*-value**	**PI at the end of learning**	***t*-value**	***p*-value**	**slope during learning**	***t*-value**	***p*-value**	**Classification**
“naïve” (*n* = 20)	0.84 ± 0.10	N/A	N/A	1.06 ± 0.09	N/A	N/A	0.033 ± 0.034	N/A	N/A	8 shifters 12 fitters
Patient #12	0.78	−0.444	0.662	0.93	−0.203	0.841	0.038	0.144	0.887	Fitter
Patient #13	0.81	0.532	0.601	0.95	−0.171	0.866	0.013	−0.574	0.573	Fitter
Patient #15	0.99	1.419	0.172	1.40	0.561	0.581	0.059	0.775	0.448	Shifter

*Comparison of the motor performance of the three stroke patients (#12, 13, and 15) who participated in our previous tDCS study (Lefebvre et al., 2013) to the performance of the 20 other stroke patients. Analysis with the Crawford and Howell statistical test to compare an individual score to a small population did not demonstrate statistically significant difference. PI, Performance Index; N/A, not applicable*.

### Study design

Prior to the fMRI activation runs, a resting-state run was acquired except in patients #1–4 and #17. The resting-state acquisition consisted of one single 6-min run during which the patients kept their eyes closed and had to avoid moving or falling asleep.

Then, the patients performed two consecutive activation runs of motor skill learning with their paretic upper limb, using a MR-compatible mouse (NAtA Technologies, Canada). Visual feedback was projected on a screen, which was viewed via a mirror placed on the head coil. As described in a previous study (Lefebvre et al., 2012), each run (duration 8 min 41 s; 172 volumes) contained a REST condition (fixation cross) and three experimental conditions: LEARNING, EASY, and REPLAY. LEARNING required performing the motor skill learning paradigm described below. EASY required moving the cursor back and forth between two horizontal or vertical targets, without speed or accuracy constraint. This condition was designed to explore brain activation related to simple movement execution under visual control. During REPLAY, a video clip of the last LEARNING performance was displayed, and the patients were instructed to follow the cursor's displacement with their eyes without moving the hand. The REPLAY condition was designed to isolate activation related to visual and oculomotor processes.

**Figure 2:**
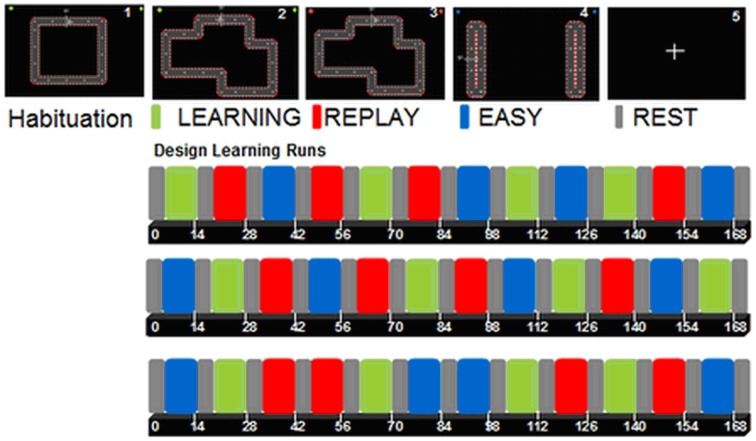
**1 simple square circuit for the habituation run (design not shown)**. 2 (green): LEARNING: subjects were instructed to move the cursor within the track as quickly and accurately as possible; 3 (red): REPLAY: subjects were instructed to follow the cursor displacement with their eyes while watching a video clip of their last LEARNING block, keeping the hands relaxed; 4 (blue): EASY: subjects were instructed to move the cursor between the two targets at a comfortable speed (50% trials with vertical movements, 50% with horizontal movements); 5: REST (gray): fixation cross.

Each condition was presented four times (30 s each; 10 volumes) during each run, and conditions were separated by four volumes (12 s) of REST (Figure 2). Beforehand, patients performed one habituation run (4 × 30 s separated by four REST volumes) of a simple circuit square.

### Motor skill learning paradigm

The motor skill learning paradigm consisted in moving a cursor controlled by an MR-compatible mouse along a complex circuit under visual feedback (Lefebvre et al., 2012, 2013). Patients were asked to complete as many laps as possible while keeping the cursor within the track (SAT) and to improve their performance over time.

### Behavioral analysis

Error was defined as the surface area between the actual trajectory and the ideal trajectory (i.e., midline of the track since the cursor had the same width as the track). Velocity was the first derivative of the position. Velocity and error during the LEARNING blocks were averaged as mean error and mean velocity over 3 s time bins [corresponding to repetition time (TR)]. To quantify motor skill learning as change of SAT, error and velocity were combined. Based on mean velocity and error, four indices were computed (for details see Lefebvre et al., 2012, 2013): error index (Pe) = constant error/patient mean error; velocity index (Pv) = patient mean velocity/constant velocity; Performance Index (PI) = Pv ^*^ Pe over bins of 3 s, then averaged for each LEARNING block; and finally Learning Index (LI) = [(PI − PI initial)/PI initial] ^*^ 100, which was computed for each LEARNING block as a percentage of the PI relative to the baseline performance during the first block of LEARNING (PI initial). Based on the LI changes over time observed previously in healthy individuals and chronic stroke patients (Lefebvre et al., 2012, 2013), motor skill learning could follow three behavioral trajectories, as described in the Introduction: shift, fit or no learning.

Student's *t*-tests were performed to determine whether baseline clinical characteristics (age, time since stroke, mRS, NIHSS, PPT, and ABILHAND) were related to the shift or fit behaviors. Similarly, Chi-square tests were calculated to investigate differences between subgroups (shifter or fitter stroke patients) based on stroke localization (cortical/subcortical), gender and whether the paretic hand was dominant or non-dominant. These comparisons were done with SigmaStat 3.5 (Systat Software San Jose, CA).

### Imaging acquisition parameters

A 3T scanner with a 32-channel head coil (Siemens Verio, Germany) was used to acquire fMRI scans of brain activity with repeated single-shot echo-planar imaging, using the following parameters: *TR* = 3000 ms, echo time (*TE*) = 23 ms, flip angle (*FA*) = 90°, matrix size = 64 × 64, field of view (FOV) = 220 × 220 mm^2^, slice order descending and interleaved, slice thickness = 2 mm (no gap) and number of slices = 59 (whole brain), voxel size = 23.8 mm^3^ (3.4375 ^*^ 3.4375 ^*^ 2). Resting-state scans were acquired via repeated single-shot echo-planar imaging with the following parameters: *TR* = 1800 ms, *TE* = 23 ms, *FA* = 80°, matrix size = 64 × 64, FOV = 240 × 240 mm, slice order ascending and interleaved, slice thickness = 3.8 mm (no gap) and number of slices = 35 (whole brain), voxel size = 53.4 mm^3^ (3.75 ^*^ 3.75 ^*^ 3.8). The whole brain was acquired 200 times (200 volumes = 6 min). The whole brain was scanned 172 times per learning run and 60 times during the habituation run. A three-dimensional (3D) T1-weighted data set covering the whole brain was acquired (1 mm^3^, *TR* = 1600 ms, *TE* = 2.39 ms, *FA* = 9°, matrix size = 512 × 512, FOV = 256 × 256 mm^3^, 176 slices, slice thickness = 1 mm, no gap).

### Pre-processing and statistical analyses

fMRI data were analyzed using BrainVoyager QX (Version 2.4.2.2070, Brain Innovation, The Netherlands). For patients with stroke lesions on the right side of the brain, both 3D-T1 and functional data were flipped. The pre-processing of the functional data consisted of a slice time scan correction, temporal high-pass filtering (removing temporal frequencies below three cycles/run) and 3D motion correction for head movements using a rigid body algorithm. If a movement >2 mm (or degrees) was observed in one of the 6 directions, the related movement parameters were using as regressors of non interest to remove potential residuals motion artifacts. Functional data were analyzed using the General Linear Model (GLM), consisting of predictors based on specific experimental conditions, in which beta weights measure the potential contribution of the predictors in each voxel time course. Coregistrations using gradient-driven affine transformations with 9 alignment parameters (3 translations, 3 rotations and 3 scales) between functional runs and 3D-T1 weighted scans of each patient were performed automatically, and then manually corrected. All anatomical and functional volumes were spatially normalized in Talairach space (Talairach and Tournoux, 1988) to allow group analysis. The statistical maps obtained were overlaid on the 3D T1-weighted scans. Functional runs were smoothed in the spatial domain with a Gaussian filter of 5 mm. The resting-state scans were not smoothed.

### fMRI processing

#### Whole-group analyses

##### Whole-brain RFX

First, a whole-group RFX analysis involving the 23 patients who achieved learning was constructed. The brain activation associated with each condition was explored at the whole-brain level using the following contrasts [LEARNING] (main condition); [EASY] (areas involved in lower aspects of movement control and execution); [REPLAY] (areas involved in visual and oculomotor activity minus REST activation); and [LEARNING - (REPLAY + EASY)]. This contrast was designed to explore the activation underlying the first stages of motor skill learning after subtracting the activation related to lower aspects of movement control and to visual-oculomotor activity (which were different between [REPLAY] and [EASY] since their respective visual feedbacks were different). Correlation analyses between the fMRI activation during [LEARNING] and the patient's clinical characteristic (the mRS score, the NIHSS score, the Abilhand score and the PPT score of the paretic hand) were performed.

##### Pearson correlations

Pearson correlation analyses were performed to identify the key area(s) where activation changes had the highest correlations with motor skill learning. For this analysis, performance (PI) values of each patient were averaged, and the correlation was performed between the eight beta weights [one averaged value for each block (mean beta for the whole group at each block)] of each ROIs with significant activation with [REPLAY], [LEARNING] and [LEARNING − (REPLAY + EASY)] and the eight PI values [one averaged value for each block (mean PI for the whole group at each block)]. The beta weights were extracted as the mean beta weight of the ROI. The correlations were done with SigmaStat 3.5.

##### Resting-state FC

For each patient (*n* = 20) an independent component analysis (ICA) extracted 40 components, using the “FastICA” algorithm implemented in BrainVoyager. Then, a self-organizing group-level ICA was used to classify the 40 components based on a clustering procedure that accepts only one component per subject in each cluster. Across these 40 clusters, five networks of interest were identified: the default-mode network (DMN) encompassing the posterior cingulate cortex, the medial prefrontal cortex, the lateral parietal cortex and the parahippocampal gyrus (Buckner et al., 2008); the somatomotor network including the primary and higher order motor and sensory areas (Biswal et al., 1995; Carter et al., 2010; Laird et al., 2011), the salience network including the anterior insula and the dorsal anterior cingulate (Seeley et al., 2007), the dorsal attentional network (DAN) including frontoparietal areas (Carter et al., 2010), and the visual network (VN) involving much of the occipital cortex (Beckmann et al., 2005).

The baseline PI values, the mRS score, the NIHSS score, the Abilhand score, the PPT score of the paretic hand and the amount of learning at the end of the session (LI) were used as covariates to assess whether the resting-state FC in the pre-mentioned networks correlated with the baseline motor performances or motor skill learning potential of the patients.

#### Subgroup (shifters/fitters) analyses

##### Whole-brain ANOVA

A whole-brain 2 factors ANOVA using condition estimates (beta values) from the first-level whole-group RFX GLM analysis constructed with 46 runs (23 stroke patients participated in a single sessions with two runs) was performed to directly compare the activation associated with the first stages of motor skill learning between each patients' subgroup [first factor: fMRI conditions (LEARNING, EASY, and REPLAY), second factor: patients' subgroup (shifters/fitters)]. The age and time since stroke were used as additional covariates.

##### ROIs comparison

The mean beta weights of the ROIs involved in the first stages of motor skill learning (identified with [LEARNING - (REPLAY + EASY)] in the whole-group RFX analysis) were used to perform Student *t*-tests to compare respective activation in these ROIs between shifter and fitter stroke patients (one averaged beta weigh per condition for each patient).

##### Separate RFX subgroup analyses

Two additional whole-brain RFX were constructed separately for each subgroup (i.e., shifters and fitters). The brain activation associated specifically with the first stages of motor skill learning was explored at the whole-brain level using the [LEARNING − (REPLAY + EASY)] contrast.

##### Subgroup Pearson correlations

Pearson correlation analyses were performed to identify the key area(s) where activation changes had the highest correlations with motor skill learning separately for each subgroup. The correlations were performed between the eight beta weights of each ROIs with significant activation with [LEARNING − (REPLAY + EASY)] and the eight PI values separately for each subgroup. The beta weights are extracted as the mean beta weights of the ROIs.

##### Resting-state FC comparison between subgroups

Finally, a two factors whole-brain ANOVA was performed to compare the five pre-specified networks between the two subgroups [first factor: resting-state networks (DMN, somatomotor network, salience network, DAN, VN), second factor: subgroups (shifters, fitters)].

## Results

### Behavioral results

**Figure 3:**
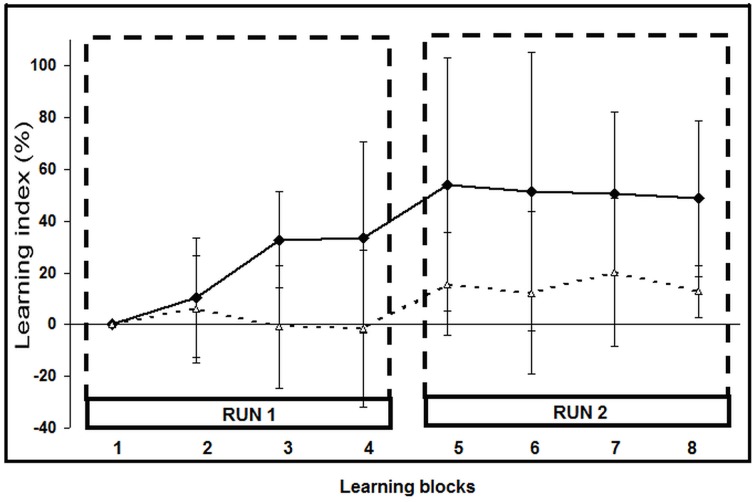
**Learning Index (LI) evolution across learning blocks**. The eight LI values (mean ± SD) correspond to the LI during each learning block. Black squares: shifter stroke subgroup (*n* = 9; LI, 49 ± 30% at the last block); white triangles: fitters stroke subgroup (*n* = 14; LI: 13 ± 10%; *p* = 0.0005).

Of the 25 stroke patients, two (#2 and 6) were classified as non-learners since their motor performance deteriorated; they were excluded from further analyses. The 23 remaining patients achieved motor skill learning: nine were classified as shifters and 14 as fitters (Figure 3; Supplementary Figure 2). At the end of the second learning run, the performance of the shifters had improved significantly more (LI: 49 ± 30%; mean ± SD) than that of the fitters (LI: 13 ± 10%; *p* < 0.001). Raw PI scores are listed in Supplementary Table 2 and SAT for each patient are illustrated in Supplementary Figure 2.

Age, time since stroke, mRS, NIHSS, PPT, and ABILHAND scores were not significantly different between the two subgroup (*p* = 0.43, 0.76, 0.66, 0.90, 0.29, and 0.73, respectively, Student's *t*-test). Finally, the two subgroups were also not different based on the type of stroke (cortical versus subcortical; *p* = 0.47, Chi-square), the gender (*p* = 0.96, Chi-square) or whether the paretic hand was dominant or non-dominant (*p* = 0.18, Chi-square).

### fMRI results

#### Whole-group analysis

##### Whole-group RFX analysis

**Figure 4:**
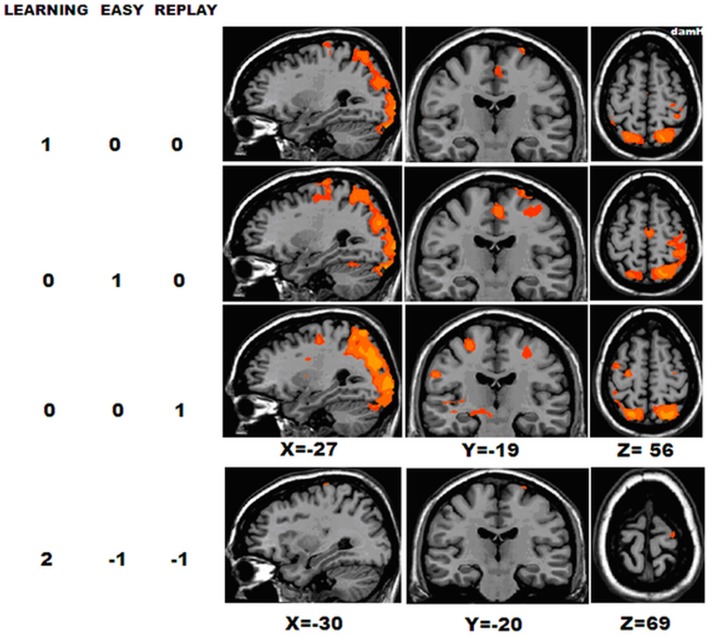
**Whole-group activation**. BOLD activation for the 23 chronic stroke patients in the following contrasts, [LEARNING], [REPLAY], and [EASY] [RFX *t*_(22)_ = 2.19; p_UNCORRECTED_ < 0.04], and [LEARNING − (REPLAY + EASY)] [RFX *t*_(22)_ = 2.10; p_UNCORRECTED_ < 0.04]. DamH, damaged hemisphere.

As shown in Figure 4 and Table 3, whole-group RFX analysis revealed the areas activated during each condition [*t*_(22)_ = 2.19; p_UNCORRECTED_ < 0.04]. Areas activated during LEARNING were: M1 [Brodmann Area (BA) 4] in the damaged hemisphere (M1_damH_), the dorsal premotor cortex (PMd_damH_, BA 6), SMA_damH_ (BA 6), bilateral posterior parietal cortex (PPC, BA 7), bilateral primary somatosensory cortex (S1, BA 3), DLPFC_damH_ (BA 9) and bilateral visual cortex. Areas activated during EASY were: M1_damH_, PMd_damH_, bilateral SMA, bilateral PPC, inferior parietal cortex (IPC_damH_, BA 40), S1_damH_, bilateral anterior cerebellum and bilateral visual cortex. As expected, the intense visual area activations during LEARNING were also observed in REPLAY, as well as activation in bilateral ventral premotor cortex (PMv), bilateral PMd, thalamus_undamH_, bilateral putamen, bilateral PPC and IPC. No significant correlation was observed between the fMRI activation during LEARNING and the baseline clinical characteristic of the patients.

**Table 3:** **Whole-group RFX analysis**.

**Brain area**	**BA**	**Mean *x***	**Mean *y***	**Mean *z***	**mm^3^**	**Activation peak**		**Correlations between PI and beta weights (*r*-values)**
						***t*-value**	***p*-value**	
**LEARNING**								
SMA_damh_	6	−4	−17	50	439	2.95	0.007	0.00
M1_damh_	4	−31	−28	59	1348	3.47	0.002	0.11
PMD_damh_	6	−25	−19	61	199	3.09	0.005	0.70
S1_damh_	3	−35	−40	57	342	3.58	0.02	0.01
S1_undamh_	3	39	−40	57	111	2.88	0.009	0.11
PPC_damh_	7	−20	−67	49	10,546	5.30	<0.0005	−0.16
PPC_undamh_	7	17	−69	48	8397	5.38	<0.0005	−0.16
DLPFC_damh_	9	−40	41	32	208	2.98	0.007	−0.82
Visual areas_damh_	18–19	−29	−85	4	11,681	5.71	<0.0005	0.25
Visual areas_undamh_	18–19	30	−82	4	20,101	5.88	<0.0005	0.06
**EASY**								
SMA_damh_	6	−4	−17	52	1386	4.61	<0.0005	^**^
SMA_undamh_	6	2	−14	52	743	4.14	<0.0005	
M1_damh_	4	−32	−28	56	4017	4.23	<0.0005	
PMD_damh_	6	−31	−16	51	2877	3.57	0.002	
S1_damh_	3	−35	−38	55	1253	3.94	0.0007	
PPC_damh_	7	−21	−64	50	13,775	5.75	<0.0005	
PPC_undamh_	7	17	−69	47	7768	5.04	<0.0005	
IPC_damh_	40	−43	−40	53	1802	3.94	0.0007	
DLPFC_damh_	9	−40	42	31	528	3.92	0.0007	
Cerebellum ipsilateral to paretic hand		11	−47	−15	989	3.81	0.001	
Cerebellum ipsilateral to non−paretic hand		−28	−56	−20	625	3.13	0.005	
Visual areas_damh_	18–19	−30	−82	3	19,597	5.15	<0.0005	
Visual areas_undamh_	18–19	30	−79	2	31,557	7.25	<0.0005	
**REPLAY**								
PMd_damh_	6	−39	−7	36	2414	5.06	<0.0005	−0.006
PMd_undamh_	6	39	−7	42	3262	4.12	<0.0005	0.25
PMv_damh_	6	−55	2	37	609	5.67	<0.0005	0.21
PMv_undamh_	6	45	−5	43	1898	4.12	<0.0005	0.14
PPC_damh_	7	−23	−68	39	26,185	7.20	<0.0005	0.09
PPC_undamh_	7	24	−72	34	29,613	7.16	<0.0005	0.05
IPC_damh_	40	−34	−41	58	187	3.32	0.003	0.09
IPC_undamh_	40	37	−41	57	104	3.34	0.003	0.05
Thalamus_undamh_		26	−27	0	1890	5.20	<0.0005	−0.02
Putamen_damh_		−19	−10	10	736	4.22	<0.0005	−0.09
Putamen_undamh_		28	−5	−1	3151	4.33	<0.0005	0.13
Visual areas_damh_	18–19	−33	−79	−4	35,428	6.22	<0.0005	0.07
Visual areas_undamh_	18–19	31	−78	−6	3753	6.91	<0.0005	0.25
**[LEARNING − (REPLAY + EASY)]**								
PMd_damH_	6	−25	−20	69	42	2.34	0.029	0.71
M1_damH_	4	−31	−26	66	44	2.29	0.031	0.12

*Whole-group RFX analysis for the [LEARNING], [EASY], [REPLAY] contrasts, [t_(22)_ = 2.19; p_UNCORRECTED_ < 0.04], and the [LEARNING − (REPLAY + EASY)] contrast [t_(22)_ = 2.10; p_UNCORRECTED_ < 0.04]. BA, Brodmann area; SMA, supplementary motor area; M1, primary motor cortex; PMd, dorsal premotor cortex; S1, primary somatosensory cortex; damH, damaged hemisphere; undamH, undamaged hemisphere; mm^3^, activated volume (equivalent to the number of activated anatomical voxels since voxels were isotropic (1 mm^3^), see the Material and Methods Section)*.

** *No correlation have been done between the PI and the beta weights in the ROIs activated during [EASY]*.

Next, to focus on the areas involved in the first stages of motor skill learning, the [LEARNING − (REPLAY + EASY)] contrast was computed [*t*_(22)_ = 2.10; p_UNCORRECTED_ < 0.04], it showed significant activation in the M1_damH_ (44 mm^2^) and PMd_damH_ (42 mm^2^).

##### Pearson correlation analyses

At the whole-group level, correlation analyses between the PI and beta weights of each ROI activated in [LEARNING] in the whole-group RFX analysis, showed a statistically significant positive correlation in the PMd_damH_ (*r* = 0.70, *p* = 0.05) and a negative correlation in the DLPFC_damH_ (*r* = −0.82, *p* = 0.01). Correlations in the other areas activated in [LEARNING] (i.e., M1_damH_, SMA_damH_, bilateral S1, bilateral PPC, and bilateral visual cortical areas) were not statistically significant (*r* ranging between −0.16 and +0.25).

Correlation analyses performed based on [LEARNING − (REPLAY + EASY)] showed a statistically significant correlation exclusively in the PMd_damH_ (*r* = 0.71, *p* = 0.048). It is worth noting that correlation analyses between the PI and beta weights of each ROI activated in [REPLAY] did not demonstrate any statistically significant correlation.

##### Resting-state FC

**Figure 5:**
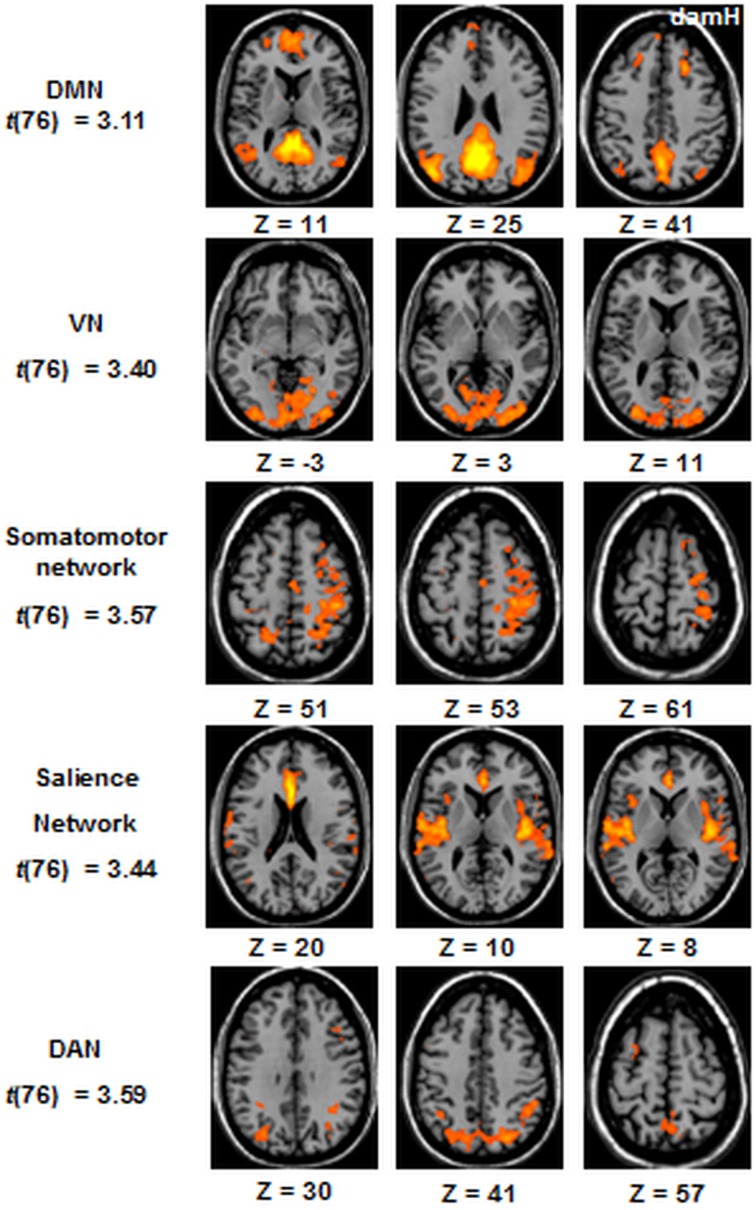
**ICA resting-state networks at the whole-group level**. Statistical maps are thresholded at (qFDR) < 0.05. DMN, default mode network; VN, Visual Network; DAN, dorsal attentional network. DamH, damaged hemisphere.

The resting-state FC was obtained for the 5 following networks at the whole-group level (*n* = 20): the DMN, the DAN, the VN, the somatomotor network, and the salience network [Figure 5, (qFDR) < 0.05, *t*_(76)_].

No significant correlation were observed in any of these five networks between the resting-state FC and the baseline clinical characteristic of the patients or the amount of LI at the end of the session.

#### Subgroup (shifters/fitters) analysis

##### Whole-brain ANOVA

The whole-brain ANOVA revealed a significant effect of the two factors and their interaction [*fMRI conditions*: *F*_(2, 42)_ = 6.27, q(FDR, False Discovery Rate) = 0.05; *patients' subgroup*: *F*_(1, 21)_ = 6,27, *p* = 0.02; *interaction*: *F*_(2, 42)_ = 6,27, *p* = 0.004].

The *post-hoc* analyses contrasted the patients' subgroup for each fMRI condition. There was no significant difference between the two patients' subgroup for [EASY] [*t*_(44)_ = 3.00; q(FDR) = 0.05] and for [REPLAY] [*t*_(44)_ = 3.00]. With [LEARNING], in the fitters subgroup 23 voxels in the posterior parietal cortex of the damaged hemisphere [PPC_damH_, BA 7, (mean *x* = −17, mean *y* = −66, mean *z* = 61)] were significantly more activated; in the shifters subgroup 16 voxels in the cerebellum ipsilateral to the paretic hand (mean *x* = 38, mean *y* = −37, mean *z* = −27) were significantly more activated [*t*_(44)_ = 3.00].

##### ROIs comparison

In each ROI significantly activated, Student *t*-tests were performed to compare the BOLD activation between shifter and fitter patients for [LEARNING − (REPLAY + EASY)]. These comparisons, summarized in Table 4 and in Supplementary Table 3 (for additional contrasts), showed a statistically significant differences exclusively in the PMd_damH_ (shifters > fitters, p_*Bonferroni−corrected*_ = 0.02) and a non-significant trend in PMd_undamH_ (shifters > fitters, p_*Bonferroni−corrected*_ = 0.15).

**Table 4:** **Differential activation between “fitter” and “shifter” stroke patients (whole-group RFX analysis)**.

**Brain area**	**Contrasts**			**Beta weights (mean ± SD)**		**Student *t*-test**
	**LEARNING**	**EASY**	**REPLAY**	**“Shifters”**	**“Fitters”**	***p*-value *Bonferroni-corrected***
PMd_damH_	2	−1	−1	0.90 ± 0.86	−0.003 ± 0.57	0.02
PMd_undamH_	2	−1	−1	0.39 ± 0.33	0.002 ± 0.42	0.15
SMA_damH_	2	−1	−1	0.34 ± 0.55	0.03 ± 0.58	0.99
M1_damH_	2	−1	−1	0.31 ± 0.78	0.14 ± 0.60	0.99
S1_damH_	2	−1	−1	0.23 ± 0.81	0.04 ± 0.59	0.99

*SMA, supplementary motor area; M1, primary motor cortex; PMd, dorsal premotor cortex; S1, primary somatosensory cortex; damH, damaged hemisphere; undamH, undamaged hemisphere*.

##### Separate RFX subgroup analyses

**Figure 6:**
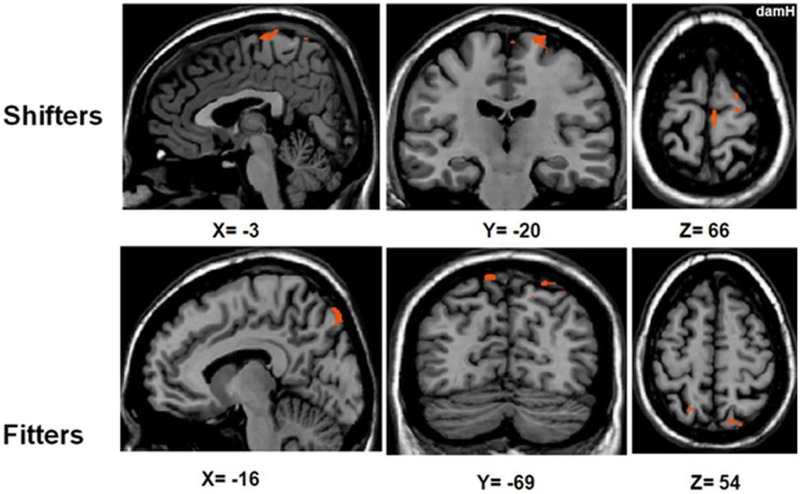
**Activation in the shifters and fitters stroke subgroups**. BOLD activation for [LEARNING − (REPLAY + EASY)] for shifter stroke patients [RFX *t*_(8)_ = 2.31; p_UNCORRECTED_ < 0.05] and fitter stroke patients [RFX *t*_(13)_ = 2.17; p_UNCORRECTED_ < 0.05]. Note that the activation in the shifter stroke subgroup is distributed in a sensorimotor/premotor network (SMA, bilateral PMd, M1_damH_, and S1_damH_), whereas significant activation in the fitters stroke subgroup is limited to the bilateral PPC. DamH, damaged hemisphere.

To compare the activation patterns in the shifter and fitter patients, separate RFX subgroup analyses were computed with [LEARNING − (REPLAY + EASY)] (Figure 6, Table 5). In the shifters subgroup [*t*_(8)_ = 2.31; p_UNCORRECTED_ < 0.05], five areas were activated: the SMA, bilateral PMd, M1_damH_, and S1_damH_. In the fitters subgroup [*t*_(13)_ = 2.17; p_UNCORRECTED_ < 0.05], only the bilateral PPC was significantly activated.

**Table 5:** **RFX subgroup analysis with [LEARNING − (REPLAY + EASY)]**.

**Brain area**	**BA**	**Mean *x***	**Mean *y***	**Mean *z***	**mm^2^**	**Activation peak**		**Correlations between PI and beta weights**	
						***t*-value**	***p*-value**	***r*-value**	***p*-value *Bonferroni-corrected***
**SHIFTERS**									
SMA_damH_	6	−4	−24	67	98	3.93	0.004	0.29	0.99
PMd_damH_	6	−18	−18	68	109	3.20	0.013	0.91	0.01
PMd_undamH_	6	15	−17	72	10	2.48	0.038	0.79	0.1
M1_damH_	4	−10	−29	68	64	3.20	0.013	0.28	0.99
S1_damH_	3	−43	−35	53	18	3.05	0.016	−0.22	0.99
**FITTERS**									
PPC_damH_	7	−16	−71	51	287	2.81	0.015	0.43	0.60
PPC_undamH_	7	17	−65	55	107	3.45	0.004	0.19	0.99

*BA, Brodmann area; SMA, supplementary motor area; M1, primary motor cortex; PMd, dorsal premotor cortex; S1, primary somatosensory cortex; PPC, posterior parietal cortex; damH, damaged hemisphere; undamH, undamaged hemisphere; mm^3^, activated volume (equivalent to the number of activated anatomical voxels since voxels were isotropic (1 mm^3^), see Material and Methods). Shifter stroke subgroup RFX at t_(8)_ = 2.31; p_UNCORRECTED_ < 0.05; fitter stroke subgroup RFX at t_(13)_ = 2.17; p_UNCORRECTED_ < 0.05*.

Analyses based on additional areas classically involved in motor skill learning but not revealed in the present study were performed (e.g., in healthy volunteers: the cerebellum ipsilateral to the working hand, (Lefebvre et al., 2012); in stroke patients: the DLPFC_damH_, (Meehan et al., 2011); and the striatum_damH_, Bosnell et al., 2011). No significant difference in the beta weights between shifters and fitters subgroups was found in the cerebellum [ROI from (Lefebvre et al., 2012): *x* = −21, *y* = −45, *z* = −21, 73 mm^3^; *p* = 0.39], nor in the DLPFC_damH_ (ROI from whole-group analysis of [LEARNING]: *x* = −40, *y* = 42, *z* = 30, 363 mm^3^; *p* = 0.24), nor in the striatum_damH_ (ROI sphere of 3 mm centered on *x* = −26, *y* = 7, *z* = 5). Ten patients were excluded from this analysis centered on the striatum_damH_, since their stroke involved part of the striatum (#3, #8, #12, #13, #14, #17, #21, #22, #23, #24).

##### Subgroup Pearson correlation analyses

Based on the RFX in the shifters subgroup, the correlation analysis between the PI and beta weight of each ROI activated in [LEARNING − (REPLAY + EASY)] revealed a significant correlation exclusively in the PMd_damH_ *r* = 0.91, p_*Bonferonni−corrected*_ = 0.01) and a non-significant trend in the PMd_undamH_ (*r* = 0.79, p_*Bonferonni−corrected*_ = 0.1). In the fitters subgroup [*t*_(13)_ = 2.17; p_UNCORRECTED_ < 0.05], only the bilateral PPC was significantly activated but there was no significant correlation between the PI and beta weight changes.

In both the fitters and shifters subgroups, in each area where a significant correlation between the beta weight and the PI was found, there was also a positive correlation with the velocity's change and a negative correlation with the error's change. No areas showed correlations exclusively with error or velocity change.

#### Resting-state FC comparison between subgroups

**Figure 7:**
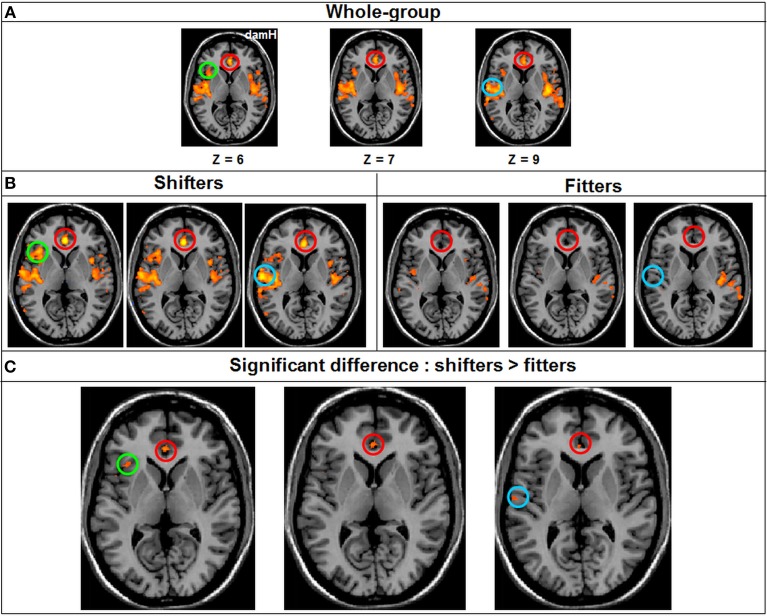
**Resting-state FC Whole-brain ANOVA. (A)** FC in the salience network in the Whole-group (qFDR < 0.05), **(B)** FC in the salience network in each subgroup (qFDR < 0.05). **(C)** The result of the whole-brain ANOVA (qFDR < 0.05), comparing the shifters with the fitters subgroups in the salience network, with higher FC in the shifter subgroup. DamH, damaged hemisphere; red circle, anterior cingulate cortex (BA 24); green circle, insula (BA 13); blue circle, temporal lobe (BA 42).

A whole-group ANOVA was created to compare the FC between the two patients' subgroup [shifters (*n* = 9)/fitters (*n* = 11)]. At qFDR < 0.05 for the DMN, the VN, the DAN and the somatomotor network no statistically significant differences were observed between the two patients' subgroup. By contrast, there was a statistically significant difference for the salience network where the FC was significantly higher in the shifters compared with the fitters subgroup [qFDR < 0.05; *t*_(76)_ = 4.78] especially in the anterior cingulate cortex (BA 24; 124 voxels; *x* = 0, *y* = 34, *z* = 7), insula [BA 13; 39 voxels; *x* = 35, *y* = 20, *z* = 5] and temporal lobe [BA 42; 26 voxels; *x* = 53, *y* = −13, *z* = 10] (Figure 7).

## Discussion

In a group of 23 chronic stroke patients, the first stages of motor skill learning with the paretic upper limb were associated with a bilateral fMRI activation pattern characterized by a positive correlation in the PMd_damH_ and a negative correlation in the DLPFC_damH_. After subtracting activation related to visual/visuomotor processes and lower aspects of motor control, correlation with motor skill learning was restricted to the PMd_damH_. The FC acquired with resting-state fMRI before the learning runs showed correlation neither with the baseline motor performance nor with the amount of motor skill learning. Comparison between the fitters and shifters stroke subgroups revealed two distinct brain activation patterns. In the less efficient fitters, significant fMRI activation was restricted to the bilateral PPC but did not correlate with motor skill learning. In contrast, in the more efficient shifters, fMRI activation encompassed the bilateral PMd, SMA_damH_, M1_damH_ and S1_damH_. The bilateral PMd, especially PMd_damH_, showed the most significant correlation between fMRI activation changes and the first stages of motor skill learning in shifters. In the resting-state salience network, the FC measured before learning was higher in the shifters subgroup; no significant difference was found in the other resting-state networks.

### Learning a motor skill with the paretic upper limb

A progressive worsening of motor performance was observed in two patients (8%). There were no obvious differences in demographic or stroke characteristic between these two non-learners and the other patients. We speculate that an excessive fatigue or a lack of motivation may have played a role, but these aspects were not assessed. Alternatively, in these individuals, strokes may have destroyed key areas that support motor skill learning. However, this seems unlikely for two reasons. First, their strokes were similar in extent and location to those of the 23 other patients. Second, to date, there is no compelling evidence that a specific brain injury may abolish motor skill learning with the paretic upper limb. Indeed, stroke patients remain able to learn new motor skills (Platz et al., 1994; Carey et al., 2002; Boyd et al., 2010; Meehan et al., 2011) and motor skill learning is impaired rather than abolished after a focal lesion of the basal ganglia (Vakil et al., 2000; Exner et al., 2001) or prefrontal cortex (Gomez Beldarrain et al., 2002).

### Reorganized motor skill learning network after stroke

In chronic hemiparetic stroke patients, the evolution of the beta weights across the two runs correlated positively with successful (early) motor skill learning in the PMd_damH_ and negatively in the DLPFC_damH_. In healthy individuals, we previously found a (positive) correlation only in the SMA (Lefebvre et al., 2012). The visual comparison of fMRI activations between non-age matched healthy individuals and hemiparetic stroke patients shown obvious pattern differences (see Supplementary Materials, and Supplementary Figure 3). Although the SMA_damH_ was also recruited in chronic hemiparetic stroke patients, the PMd_damH_ was the key area correlating with the first stages of motor skill learning, suggesting a neuroplastic reorganization. Previous motor skill learning studies in stroke patients led to conflicting observations about PMd. After extensive tracking training, performance improvement was associated with an increased activation in PMd_damH_ at the expense of PMd_undamH_ (Carey et al., 2002). In another study, activation in PMd_undamH_ correlated with motor sequence accuracy at retention (Meehan et al., 2011). The current study clearly showed that PMd_damH_ plays a key role during the early stage of learning a motor skill involving a SAT with the paretic hand. This - likely compensatory - activation in PMd_damH_ is coherent with the observations that PMd (i) is crucial for motor skill learning in healthy individuals (Kantak et al., 2012; Hardwick et al., 2013), (ii) is involved in motor function recovery after stroke (Carey et al., 2002; Fridman et al., 2004; Tombari et al., 2004; Lotze et al., 2006; Bestmann et al., 2010; Schulz et al., 2012), and (iii) is part of the reorganized network involved in stroke patients performing simple motor tasks (Johansen-Berg et al., 2002; Calautti et al., 2010).

A recent study in stroke patients showed strong compensatory activation of the bilateral DLPFC and prefrontal BA 46 in the undamaged hemisphere during motor sequence learning and of the prefrontal BA 8 in the undamaged hemisphere during the retention test (Meehan et al., 2011). In the current study, activation in the DLPFC_damH_ correlated *negatively* with performance improvement during the first stages of motor skill learning. This may suggest an early, transient recruitment of prefrontal attentional areas (here, DLPFC_damH_) in parallel with rising activation of the (pre-)motor network in stroke patients.

During REPLAY, intense activation was observed in the visual areas, as well as widespread activation including the bilateral PMv, PMd, putamen, PPC, IPC, and the thalamus_undamH_. However, there was no correlation between fMRI activation in these areas and motor skill learning, demonstrating that REPLAY was not involved in learning this skill through rehearsal or memory retrieval.

During the simple motor condition EASY, the bilateral cerebellum was activated, consistently with its role (i) in motor control (Sakai et al., 1998; Miall et al., 2001) and (ii) in recovered motor function after stroke (Small et al., 2002). In contrast, during motor skill learning, there was neither significant activation nor correlation within the cerebellum. The heterogeneity of the strokes might have prevented reliable recruitment of the cerebellum. However, this seems unlikely since consistent cerebellar activation was observed during simple motor performance (EASY). It is worth noting that no change in cerebellar activation associated with motor learning was reported in previous whole-brain studies in subcortical stroke patients (Bosnell et al., 2011; Meehan et al., 2011). We speculate that, in contrast to healthy individuals, this might reflect a preferential or more consistent recruitment of the premotor and/or prefrontal areas rather than the cerebellum during the first stages of motor skill learning.

### Post-stroke neuroplasticity or ageing-related modifications?

Could the observed activation pattern reflect ageing-related reorganization, rather than post-stroke neuroplasticity? Previous studies demonstrated a modification of the motor control/execution network associated with ageing (Mattay et al., 2002; Heuninckx et al., 2008). It is thus logical to infer that ageing would also influence the motor skill learning network, even though such a reorganization has yet not been formally demonstrated (Daselaar et al., 2003). Furthermore, studies comparing fMRI activation between stroke patients and age-matched healthy individuals consistently found striking differences suggestive of post-stroke neuroplastic reorganization, both for motor skill learning (Carey et al., 2002; Bosnell et al., 2011; Meehan et al., 2011) and motor performance (Zemke et al., 2003; Ward and Frackowiak, 2006; Schaechter and Perdue, 2008). This clearly demonstrates that the impact of stroke on the reorganization of brain activation is considerably greater than the impact of ageing. In addition, an external multiple regression analysis (data not shown) combining the healthy individuals (Lefebvre et al., 2012) and stroke patients did not demonstrate a statistically significant correlation between age (18–82 years old) and fMRI activation in all the activated ROIs with [LEARNING] (M1, SMA, PMd, S1, PPC, thalamus, DLPFC and cerebellum). Therefore, we conclude that the observed differences in fMRI patterns between chronic stroke patients and healthy individuals (Lefebvre et al., 2012) predominantly reflect post-stroke neuroplastic reorganization rather than ageing. In other words, chronic stroke patients recruited a reorganized (likely compensatory) network to achieve motor skill learning.

### Differential brain activation in shifter and fitter stroke patients

As in healthy individuals under similar experimental conditions (Lefebvre et al., 2012), the chronic stroke patients could be classified as shifters, fitters or non-learners. In a previous study using the same motor skill learning paradigm, more chronic stroke patients behaved as fitters rather than as shifters after 30 min of motor skill learning under control condition (sham transcranial direct current stimulation) (Lefebvre et al., 2013). These observations suggest that this motor skill learning paradigm based on a SAT allows a fine dissection of behavioral trajectories in healthy individuals and stroke patients. Demographic factors, stroke characteristics [time since stroke or stroke localization (cortical vs. subcortical lesion)] and level of impairment did not predict whether stroke patients would behave as shifters or fitters. It remains to be determined whether a genetic background, such as a polymorphism of the brain-derived neurotrophic factor (BDNF) (Bath and Lee, 2006; Kleim et al., 2006; McHughen et al., 2010), underlies these different behaviors.

In the shifter stroke subgroup, activation was found in the bilateral PMd, SMA_damH_, M1_damH_, and S1_damH_; and a significant correlation between motor skill learning and fMRI activation changes was found exclusively in the bilateral PMd, especially PMd_damH_. This suggests that the shifter stroke patients recruited neuronal resources from both hemispheres but predominantly from the damaged hemisphere to achieve efficient motor skill learning. The stronger involvement of the bilateral PMd (especially PMd_damH_) compared with the SMA_damH_ (strongly involved in healthy shifters, see Supplementary Materials) likely reflects a neuroplastic reorganization of the motor skill network. Finally, the whole-brain ANOVA demonstrated a higher level of activation in the cerebellum ipsilateral to the paretic hand in the shifters compared with fitters, suggesting a more consistent recruitment of the cerebellum in the more efficient shifter stroke patients.

As previously observed in healthy fitters (Lefebvre et al., 2012), fitter stroke patients seemed unable to efficiently or consistently engage the motor learning network observed in shifter stroke patients. Here, significant activation was found exclusively in the bilateral PPC and did not correlate with motor skill learning. Furthermore, the whole-brain ANOVA confirmed that the PPC_damh_ was more activated in the fitters stroke subgroup compared with the shifters subgroup. The PPC is involved in the planning of visuomotor tasks (Desmurget et al., 1999; Torres et al., 2013). This may suggest that the fitter stroke patients were focused (or jammed) on the visuospatial aspects of the task, possibly reflecting a protracted exploratory phase.

The current fMRI results shed new light on a previous observation in chronic stroke patients. Compared with sham, tDCS applied bilaterally over M1 (dual-tDCS) enhanced motor skill learning in 100% of chronic stroke patients (*n* = 18) (Lefebvre et al., 2013). In sharp contrast, after sham dual-tDCS, 44% of the stroke patients (*n* = 8) showed performance worsening (non-learners). Given the spread of the direct current delivered through tDCS electrodes centered over M1, it is possible that the bilateral PMd were also stimulated. If bilateral PMd are indeed crucial for efficient motor skill learning with the paretic hand in chronic stroke patients, as suggested by the current fMRI observation, stimulation of PMd could partially explain the enhancement of motor skill learning and long-term retention by dual-tDCS in stroke patients (Lefebvre et al., 2013).

### Baseline resting-state FC

In contrast with previous studies which showed that resting-state FC correlates with residual motor function in chronic stroke (Carter et al., 2010; Yin et al., 2012), no correlation between the level of motor performance and the baseline FC in any of the five resting-state networks of interest was observed in the current study. As discussed in the limitations section, this might be related to the heterogeneity of our chronic stroke patients and the smaller sample (*n* = 20) for resting-state fMRI. The choice of the outcome measure (scales) may also be of key importance when looking for correlation between resting-state FC and residual functions in chronic stroke patients.

Previous studies suggested that resting-state-fMRI FC might also be of interest for predicting the recovery potential after stroke (Park et al., 2011, 2014; Golestani et al., 2013; Ovadia-Caro et al., 2013; Dacosta-Aguayo et al., 2014). In the current study, the only correlation between baseline resting-state FC and subsequent motor skill learning was observed in the salience network where FC was higher in the shifters subgroup compared with the fitters subgroup. No difference was observed in the others resting-state networks. Thus, a higher (i.e., supposed “better”) resting-state FC within the salience network before training may predispose chronic stroke patients to learn more efficiently a new motor skill with a SAT. Since the salience network is involved in the detection of pertinent stimuli to perform a task and in the coordination of the neural resources needed to achieve this task (Uddin, 2015), one may hypothesize that the salience network plays a key role in recruiting the attentional resources that facilitate learning. The anterior insula triggers attentional shift to salient event in the anterior cingulate cortex which in turn modulates the activation of PMd (Menon and Uddin, 2010). One could thus hypothesize that the higher connectivity in the anterior cingulate cortex and anterior insula in the shifter stroke patients could favor the early recruitment of PMd during motor skill learning. This observation needs to be confirmed in a larger stroke cohort. The lack of significant correlation within the other resting-state networks may be due to the already discussed limitation. Furthermore, it is also possible that this motor skill learning session was too short, compared with the longitudinal recovery driven by neurorehabilitation (Park et al., 2011, 2014; Golestani et al., 2013; Ovadia-Caro et al., 2013; Dacosta-Aguayo et al., 2014).

### Limitations of the study

This study had four main limitations. First, patients were relatively heterogeneous in terms of stroke localization (cortical, subcortical, brainstem) and etiology (large arteries, lacunar infarcts, intracerebral hemorrhages). This heterogeneity might have enhanced the inter-individual variability and dampened the significance of the activation and resting-state BOLD signals. However, this relative heterogeneity may also be considered to be a strength, since this cohort represents real-life hemiparetic stroke patients.

Second, for all the RFX analyses (excepted for the whole-brain ANOVA), a liberal threshold of p_UNCORRECTED_ < 0.04 was applied. This threshold was chosen given the heterogeneity of the stroke population and the variability associated with large brain lesions. Due to this variability and to the technical difficulties associated with stroke patients, the level of statistical significance warranted in a population of healthy individuals is difficult to achieve, unless the recruitment is restricted to a subpopulation, typically patients with slight impairment and a single subcortical stroke. Nevertheless, the results described at group level were also confirmed at the individual level (individual correlations, paired *t*-test ROIs comparison) and were correlated with the behavioral performance evolution. Thus, even if these results have to been confirmed, they are both informative and relevant for the field.

Third, the results of the correlation analyses presented here are preliminary and need to be confirmed in a larger cohort of stroke patients. It has however to be noted that compared to the previous studies exploring motor skill learning in stroke patients (n = 9 or 10), a larger sample (*n* = 23; i.e., 25 − 2) was involved in the current study.

Fourth, a formal comparison with a group of age-matched control subjects has not been performed. However, as discussed previously, the reorganization of the motor skill learning network we observed in stroke patients is coherent with previous studies showing post-stroke compensatory reorganization of the motor control network in aged patients (Carey et al., 2002; Jaillard et al., 2005). Further studies are required to unveil the full dynamic evolution of motor skill learning in stroke patients compared with healthy controls.

## Conclusions

Compared with previous studies based on ROI analyses and/or smaller cohorts of subcortical stroke patients, the current study represents several important advances. First, the use of whole-brain fMRI analysis unveiled a network dynamically engaged during the first stages of motor skill learning in chronic hemiparetic stroke patients. Second, the application of RFX analyses to a relatively large cohort of chronic patients with various types of stroke identified more general reorganization mechanisms which are likely shared by most hemiparetic chronic stroke patients. Third, careful behavioral dissection of this motor skill learning paradigm involving a SAT provided an unprecedented level of precision to investigate the fine neuronal mechanisms underlying the first stages of motor skill learning in stroke patients.

When learning a new visuomotor skill involving a SAT with the paretic upper limb, chronic stroke patients presented a reorganized brain activation pattern involving both hemispheres with a predominant recruitment of the damaged hemisphere. The key area underlying efficient motor skill learning was bilateral PMd and especially PMd_damH_, in contrast to the key role played by the SMA in healthy individuals. This suggests a neuroplastic, compensatory recruitment of additional areas during motor skill learning in chronic hemiparetic stroke patients. Surprisingly, the correlations between baseline resting-state FC and residual motor function or motor learning potential revealed only a higher resting-state FC in the salience network in the shifter stroke subgroup. Further explorations of FC as a biomarker of recovery potential are needed. A better understanding of the neural substrates underlying motor learning in stroke patients is a crucial step forward to design the next generation of neurorehabilitation paradigms.

### Conflict of interest statement

The authors declare that the research was conducted in the absence of any commercial or financial relationships that could be construed as a potential conflict of interest.
