# Supplementary material for: Neural substrates underlying motor skill learning in chronic hemiparetic stroke patients
Source: Front Hum Neurosci. 2015 Jun 3;9:320. doi: 10.3389/fnhum.2015.00320 (PMC4452897; doi:10.3389/fnhum.2015.00320)
Supplement: Supplementary file 1 [file Table1.DOC]

**Supplementary Table 1: correlation analyses excluding # patients 12, 13 and 15.**

| **Group** | **Contrasts** | **Areas** | **Correlation excluding patients #12, 13 & 15** |
| --- | --- | --- | --- |
| Whole-group | [LEARNING] | PMd_damH_ | r = 0.77 ; p=0.03 |
|  |  | DLPFC_damH_ | r = -0.35; p = 0.39 |
|  | [LEARNING - (REPLAY + EASY)] | PMd_damH_ | r=0.92 ; p= 0.001 |
| Shifters | [LEARNING - (REPLAY + EASY)] | PMd_damH_ | r =0.86; p= 0.006 |
|  |  | PMd_undamH_ | r= 0.76; p= 0.03 |

Additional correlation analyses were performed excluding the 3 patients who participated in a previous motor learning experiment. Excluding these 3 patients did not alter the statistical significance of the correlations in the shifters subgroup or at whole group level except for the DLPFC_damH_ correlation. This could suggest that the correlation in the DLPFC_damH_ was mostly driven by the fitters, since removing two of them resulted in the loss of statistical significance. Furthermore, excluding the 3 patients did not reveal correlation in other activated areas. In the fitters subgroup, removing these 2 patients did not change the previous results and so no significant correlation between beta values and PI were observed in any areas.
