# Supplementary material for: Neural substrates underlying motor skill learning in chronic hemiparetic stroke patients
Source: Front Hum Neurosci. 2015 Jun 3;9:320. doi: 10.3389/fnhum.2015.00320 (PMC4452897; doi:10.3389/fnhum.2015.00320)
Supplement: Supplementary file 2 [file Table2.DOCX]

**Supplementary Table 2: raw PI scores**

| **Patients** | **PI score at Baseline** | **PI score at the end of learning** |
| --- | --- | --- |
| **µ 1** | 2,049 | 2,183 |
| **µ 3** | 0,453 | 0,881 |
| **µ 4** | 1,066 | 1,261 |
| **5** | 1,124 | 2,218 |
| **7** | 0,585 | 0,760 |
| **8** | 0,919 | 1,459 |
| **9** | 0,601 | 0,813 |
| **10** | 0,462 | 0,629 |
| **11** | 0,572 | 0,609 |
| **12 *** | 0,780 | 0,929 |
| **13 *** | 0,893 | 0,949 |
| **14** | 1,100 | 1,258 |
| **15 *** | 0,996 | 1,404 |
| **16** | 0,267 | 0,291 |
| **µ 17** | 0,795 | 0,949 |
| **18** | 0,750 | 0,767 |
| **19** | 1,081 | 1,590 |
| **20** | 1,981 | 2,205 |
| **21** | 0,283 | 0,341 |
| **22** | 0,772 | 0,867 |
| **23** | 0,331 | 0,353 |
| **24** | 0,410 | 0,569 |
| **25** | 1,098 | 1,116 |
| **mean ± SD** | **0.842 ± 0.452** | **1.061 ± 0.557** |
| **µ 2 $** | **1.73** | **1.58** |
| **µ 6 $** | **1.85** | **0.91** |

The raw PI scores demonstrated that despite different level of performance at Baseline, overall the patients improved their global performance across the session (end of learning). The raw PI scores of patients #12, 13 and 15 (*) were similar to those observed in the other patients. The PI of the two non-learners patients ($) were displayed at the end of the Table. The patients for which the rs-fMRI data were not acquired are identified with a µ.
