# Supplementary material for: Neural substrates underlying motor skill learning in chronic hemiparetic stroke patients
Source: Front Hum Neurosci. 2015 Jun 3;9:320. doi: 10.3389/fnhum.2015.00320 (PMC4452897; doi:10.3389/fnhum.2015.00320)
Supplement: Supplementary file 3 [file Table3.DOCX]

**Supplementary Table 3: Comparisons between the shifter and fitter stroke patients**

| **brain area** | **contrasts** | | | **beta weights (mean ± SD**) | | **Student t-test** |
| --- | --- | --- | --- | --- | --- | --- |
|  | **LEARNING** | **EASY** | **REPLAY** | **“shifters”** | **“fitters”** | **p value** |
| PMd_damH_ | 1 | 0 | 0 | 0.67 ± 0.52 | 0.17 ± 0.56 | 0.04 * |
| PMd_undamH_ | 1 | 0 | 0 | 0.30 ± 0.31 | 0.13 ± 0.52 | 0.39 |
| SMA_damH_ | 1 | 0 | 0 | 0.35 ± 0.54 | 0.26 ± 0.44 | 0.68 |
| M1_damH_ | 1 | 0 | 0 | 0.27 ± 0.61 | 0.27 ± 0.51 | 0.99 |
| S1_damH_ | 1 | 0 | 0 | 0.25 ± 0.58 | 0.08 ± 0.43 | 0.42 |
| PMd_damH_ | 0 | 1 | 0 | 0.41 ± 0.47 | 0.26 ± 0.56 | 0.49 |
| PMd_undamH_ | 0 | 1 | 0 | 0.21 ± 0.22 | 0.16 ± 0.44 | 0.74 |
| SMA_damH_ | 0 | 1 | 0 | 0.35 ± 0.45 | 0.33 ± 0.39 | 0.90 |
| M1_damH_ | 0 | 1 | 0 | 0.32 ± 0.36 | 0.31 ± 0.50 | 0.93 |
| S1_damH_ | 0 | 1 | 0 | 0.27 ± 0.35 | 0.11 ± 0.42 | 0.35 |
| PMd_damH_ | 0 | 0 | 1 | -0.02 ± 0.29 | 0.09 ± 0.56 | 0.59 |
| PMd_undamH_ | 0 | 0 | 1 | 0.005 ± 0.26 | 0.11 ± 0.47 | 0.56 |
| SMA_damH_ | 0 | 0 | 1 | 0.01 ± 0.33 | 0.16 ± 0.43 | 0.34 |
| M1_damH_ | 0 | 0 | 1 | -0.09 ± 0.24 | 0.08 ± 0.52 | 0.35 |
| S1_damH_ | 0 | 0 | 1 | 0.003 ± 0.12 | 0.02 ± 0.34 | 0.92 |

**Supplementary Table 3:** Comparisons of the contrasts [LEARNING], [EASY] and [REPLAY] between the shifter and fitter stroke patients in the activated network (whole-group RFX analysis). For the [LEARNING] contrast, a statistically superior activation in the shifters compared with fitters was found only in PMD_damH_ (*). Levels of activation for other areas and contrasts were similar between shifters and fitters.
