# Supplementary material for: Neural substrates underlying motor skill learning in chronic hemiparetic stroke patients
Source: Front Hum Neurosci. 2015 Jun 3;9:320. doi: 10.3389/fnhum.2015.00320 (PMC4452897; doi:10.3389/fnhum.2015.00320)
Supplement: Supplementary file 4 [file SupplementaryMaterial.DOC]

**Supplementary Materials: Comparison between the stroke patients and healthy individuals from (Lefebvre et al., 2012)**

At a behavioural level, at the end of the second learning block, 20 healthy individuals reached a LI improvement of 15 ± 31 % (fitters (n=9): 9 ± 23 %, shifters (n= 11): 19 ± 36 %), and three were classified as non-learners, whereas 23 stroke patients reached a LI improvement of 27 ± 27 % (fitters (n=14): 13 ± 10 %, shifters (n=9): 49 ± 30 %); two patients were classified as non-learners. This suggests that (i) at the group level, the stroke patients involved in this study were able to achieve motor skill learning overall, and (ii) the stroke patients were more prone to achieve relatively large performance improvements on this task with their paretic hand. The shifter/fitter/non-learner classification during early motor skill learning was found in both population with a relatively different distribution between shifters (50% in healthy individuals versus 36% in stroke patients) and fitters (41% versus 56%), but not in non-learners (9% versus 8%).

When comparing the spatial distribution of fMRI activation, the network underlying motor skill learning in stroke patients did not overlap with that observed in young healthy individuals during the same task (Supplementary Figure 3). This suggests that motor skill learning in stroke patients relied on a reorganised and probably compensatory network compared with healthy individuals.

In stroke patients, correlation analyses performed based on [LEARNING - (REPLAY + EASY)] showed a statistically significant (*positive*) correlation exclusively in the PMd_damH_ (r = 0.71, p = 0.048). In order to compare the involvement of PMd in healthy individuals, a whole-group correlation was performed between the PI values and beta weight of the PMd contralateral to the working hand (PMd_cwh_). There was a non-statistically significant *negative* correlation between motor skill learning and brain activation in PMd_cwh_ of healthy individuals. (r = -0.48, p = 0.22).

Finally, in the of shifter stroke subgroup, the correlation analysis between the PI and beta weight changes over time revealed a significant (positive) correlation exclusively in the PMd_damH_ (r = 0.91, p = 0.002) and PMd_undamH_ (r = 0.79, p = 0.02). Therefore, a similar analysis was performed with the healthy shifters subgroup: The correlation between PI and beta weight in PMd_cwh_ was positive but non-significant (r = 0.27, p = 0.52); in the PMd ipsilateral to the working hand, the correlation was negative and non-significant (r = -0.21, p = 0.62).

Therefore, (i) the lack of spatial overlap between the networks observed in stroke patients and healthy individuals and (ii) the differential involvements of PMd (and SMA) in motor skill learning between the groups of stroke patients and healthy individuals suggest that the stroke patients relied on a reorganised network to achieve motor skill learning with the paretic hand.
