# Supplementary material for: Neural substrates underlying motor skill learning in chronic hemiparetic stroke patients
Source: Front Hum Neurosci. 2015 Jun 3;9:320. doi: 10.3389/fnhum.2015.00320 (PMC4452897; doi:10.3389/fnhum.2015.00320)
Supplement: Supplementary file 5 [file Image1.PDF]

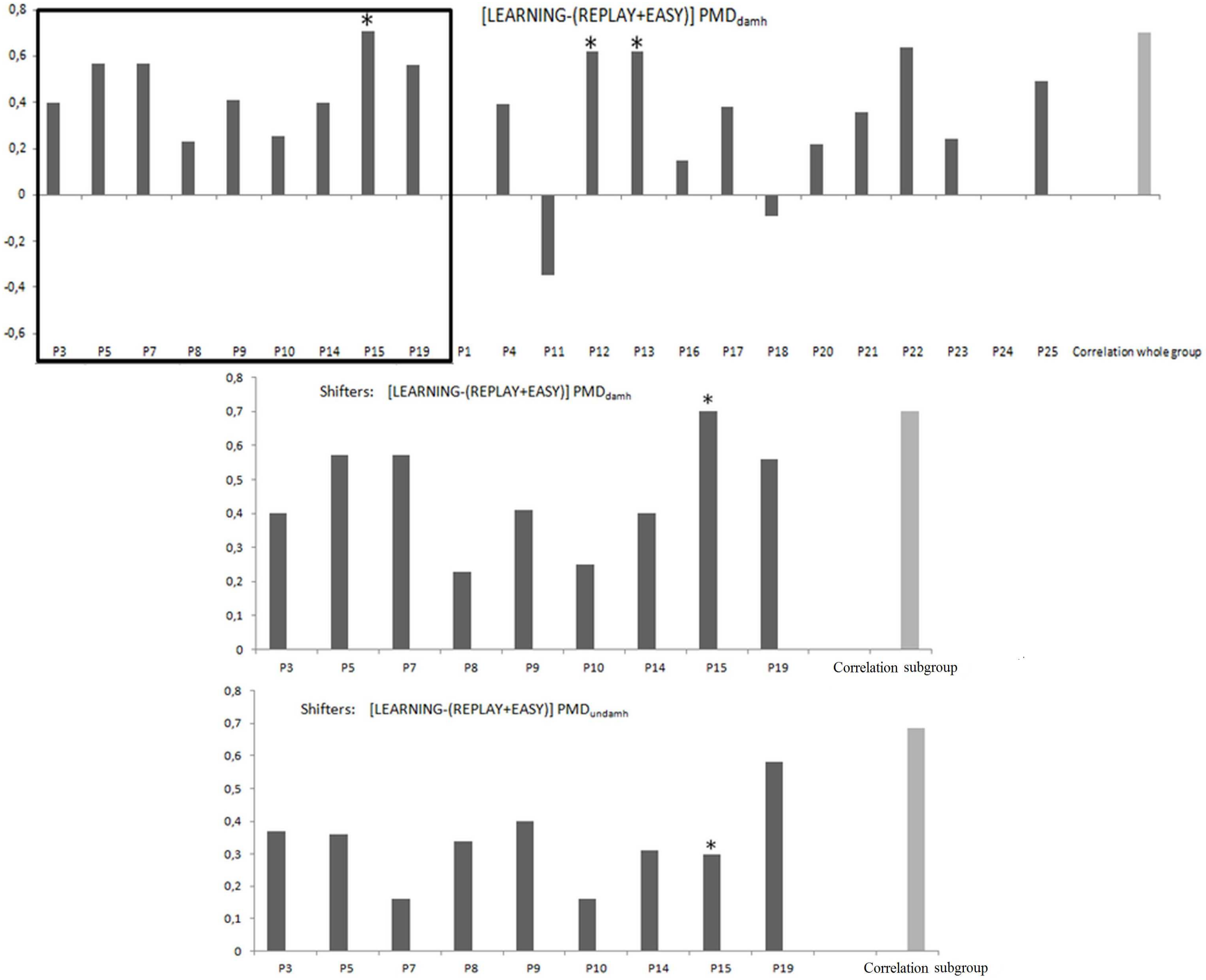

Supplementary Figure 1: Individual correlations between motor skill learning and brain activation for [LEARNING - (REPLAY + EASY)].

Upper panel. In the areas where significant correlation was observed at the whole-group level (PMD_{damH}), individual correlations were performed between the 8 PI values and 8 beta values for each patient. Note that the correlation observed in patients #12, 13 and 15 (*) were similar to those observed in the other patients. The shifter stroke patients (n=9) are identified by the large square.

Lower panel. In the areas where significant correlation was observed in the shifters group (PMD_{damH} and PMd_{undamH}), individual correlations were performed between the 8 PI values and 8 beta values for each patient.
