# Supplementary material for: Neural substrates underlying motor skill learning in chronic hemiparetic stroke patients
Source: Front Hum Neurosci. 2015 Jun 3;9:320. doi: 10.3389/fnhum.2015.00320 (PMC4452897; doi:10.3389/fnhum.2015.00320)
Supplement: Supplementary file 6 [file Image2.PDF]

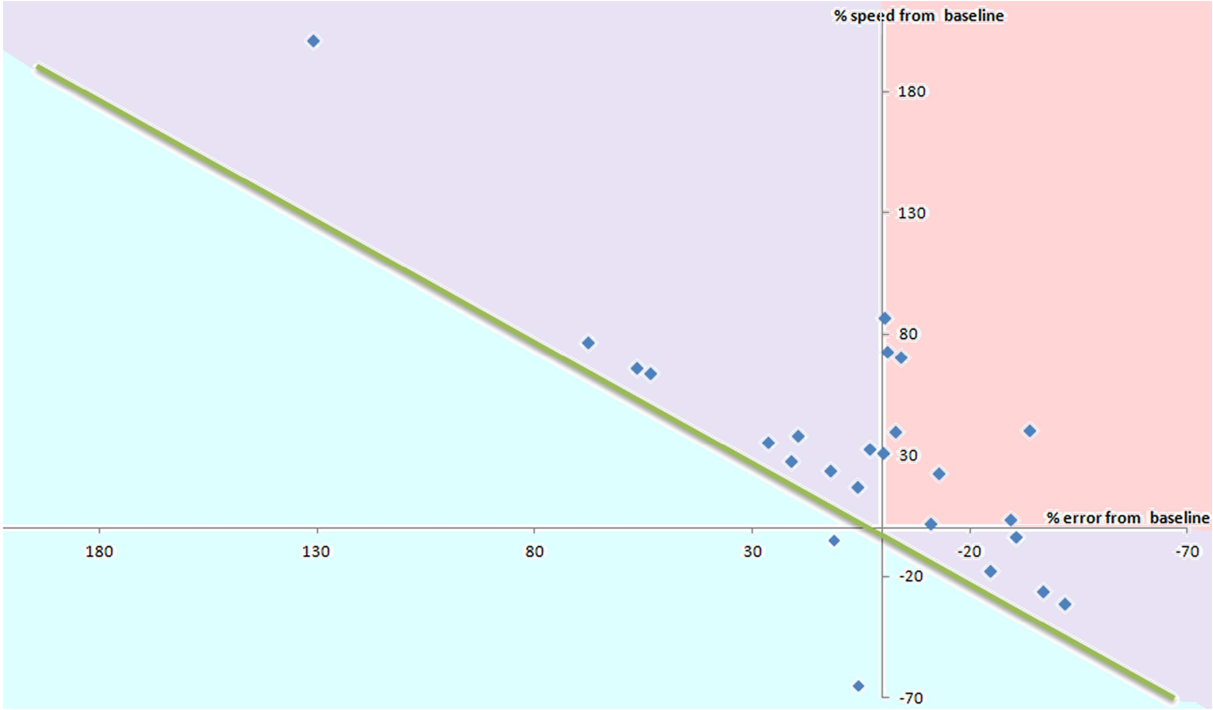

Supplementary Figure 2: Scatter plot of the SAT. In this scatter plot, the speed-accuracy trade-off (SAT) for each of the 25 chronic stroke patients is expressed as percentage change between Baseline (first block of the first run) and the last block of the second fMRI run. The ordinate axis codes velocity and the abscise axis codes error (i.e. the inverse of accuracy). The green line reflects the Fitt's law (Fitts, 1954): sliding along this line represent a strict fit with of the Fitt's law ("perfect fitter"). Below this line, motor skill learning is not achieved and motor performance worsens (deterioration or of the LI; light blue zone, n=2). Above the green line, motor skill learning is achieved since the LI is increased, either as the less efficient fit behaviour (light purple zone, n=14) or as the more efficient shift behaviour (red zone, n=9).
