# Supplementary material for: Neural substrates underlying motor skill learning in chronic hemiparetic stroke patients
Source: Front Hum Neurosci. 2015 Jun 3;9:320. doi: 10.3389/fnhum.2015.00320 (PMC4452897; doi:10.3389/fnhum.2015.00320)
Supplement: Supplementary file 7 [file Image3.PDF]

Supplementary Figure 3: Spatial extent of whole-group activation in healthy individuals and stroke patients

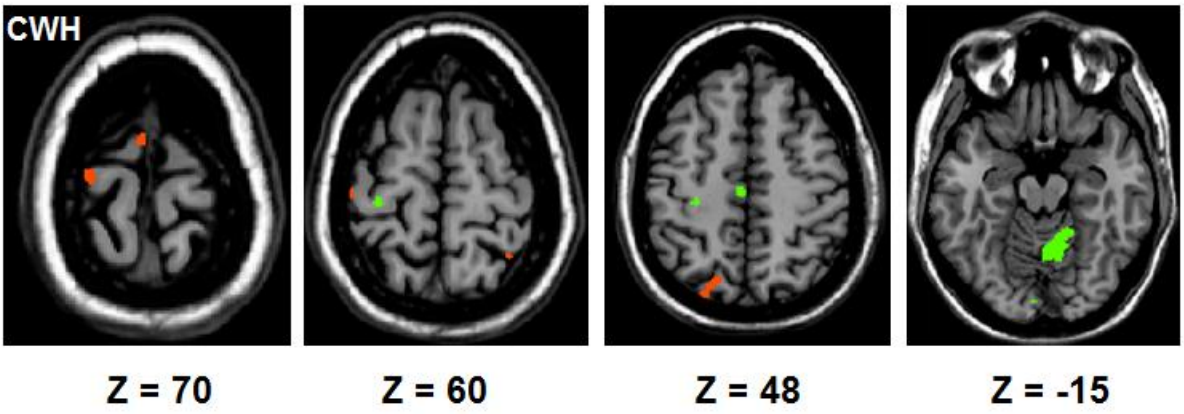

To allow for visual comparison, whole-group activation for [LEARNING - (REPLAY + EASY)] was set at a threshold of 100 voxels in M1, an area activated in both populations. Red: stroke patients, green: healthy individuals. The two networks were different and did not overlap spatially. This suggests that motor skill learning in stroke patients relied on a reorganised network compared with healthy individuals. CWH = contralateral to the working hand (i.e. paretic hand in stroke patients, non-dominant hand in healthy individuals).
